# Prevalence of β-lactamase genes, class 1 integrons, major virulence factors and clonal relationships of multidrug-resistant *Pseudomonas aeruginosa* isolated from hospitalized patients in southeast of Iran

**DOI:** 10.22038/ijbms.2019.35063.8340

**Published:** 2019-07

**Authors:** Hosein Sharifi, Gholamreza Pouladfar, Mohammad Reza Shakibaie, Bahman Pourabbas, Jalal Mardaneh, Shahla Mansouri

**Affiliations:** 1 Department of Microbiology and Virology, Kerman University of Medical Sciences, Kerman, Iran; 2 Prof Alborzi Clinical Microbiology Research Center, Shiraz University of Medical Sciences, Shiraz, Iran; 3 Kerman Infection Diseases and Tropical Medicine Research Center, Kerman University of Medical Sciences, Kerman, Iran; 4 Department of Microbiology, School of Medicine, Gonabad University of Medical Sciences, Gonabad, Iran

**Keywords:** Antibiotic resistance, Beta-lactamases, Class 1 integrons, Pseudomonas aeruginosa, RAPD-PCR, Virulence factors

## Abstract

**Objective(s):**

*Pseudomonas aeruginosa* is one of the most important nosocomial pathogens causing a high rate of mortality among hospitalized patients. Herein, we report the prevalence of antibiotic resistance genes, class 1 integrons, major virulence genes and clonal relationship among multidrug- resistant (MDR) *P. aeruginosa*, isolated from four referral hospitals in the southeast of Iran.

**Materials and Methods:**

In this study, 208 isolates of *P. aeruginosa* were collected from four referral hospitals in southeast of Iran. Disk diffusion method was used to determine susceptibility to 13 antibacterial agents. AmpC was detected by phenotypic method and β-lactamase genes, virulence genes and class 1 integrons were detected by PCR. Clonal relationship of the isolates was determined by RAPD-PCR.

**Results:**

All the isolates were susceptible to polymyxin-B and colistin. Overall, 40.4% of the isolates were MDR, among which resistance to third generation cephalosporins, aminoglycosides, and carbapenems was 47.5%, 32.3% and 40%, respectively. None of the isolates was positive for *bla*_NDM-1_ genes, while 84.5% and 4.8% were positive for the *bla*_IMP-1_ and *bla*_VIM_, metallo-β-lactamase genes, respectively. Incidence of class 1 integrons was 95% and AmpC was detected in 33% of the isolates. Prevalence of *exoA, exoS, exoU, pilB* and *nan1* were 98.8%, 44%, 26%, 8.3% and 33.3%, respectively. RAPD profiles identified four large clusters consisting of 77 isolates, and two small clusters and three singletons.

**Conclusion::**

The rate of MDR *P. aeruginosa* isolates was high in different hospitals in this region. High genetic similarity among MDR isolates suggests cross-acquisition of infection in the region.

## Introduction

*Pseudomonas aeruginosa* is a Gram-negative opportunistic pathogen and a common source of infections in hospitals, particularly in intensive care units (ICUs), where the organism infects 10-15% of the patients. Rapid increase of multidrug-resistant *P. aeruginosa* (MDRPA) isolates in clinical settings worldwide has resulted in increased mortality rate (1, 2). Several mechanisms are involved in *P. aeruginosa *resistance to antimicrobial agents such as β-lactamase production, target mutation, efflux pumps over-expression, decrease in membrane permeability and chromosomal expression of resistance encoding genes (3). Due to the pressure caused by the overuse of β-lactam antibiotics in the hospitals, various forms of β-lactamases such as the extended spectrum β- lactamases (ESBLs), AmpC and metallo-β-lactamases (MBLs) have evolved (4). Integrons are also important factors in dissemination of antibacterial resistance among different bacterial species and the association between integrons and drug resistance has been shown (5). Among the integron classes, class 1 integrons (*int*1) was the most prevalent among clinical isolates of *P. aeruginosa* in Ahvaz**, **Iran (6). In addition to antibiotic resistance, *P. aeruginosa* produces various virulence factors contributing to pathogenicity of the microorganism. Among these virulence factors are a variety of secreted proteins such as proteases, phospholipases, and exotoxin A (7). In addition, exoenzyme S is a virulence factor encoded by the *exo*S gene. It is an ADP ribosyltransferase secreted by a type-III secretion system (TTSS), delivered directly into the cytosol of epithelial cells. Another protein, exotoxin U (*ExoU*), is a necrotizing toxin with phospholipase activity, unique cytotoxic effect, capable of destroying cellular monolayers during short infection periods and plays a role in the development of septic shock (8). In some *P. aeruginosa* hospital isolates, a gene called *nan*1 encodes an extracellular neuraminidase that is responsible for the adherence to the respiratory tract and facilitates long term infection in cystic fibrosis patients (9). In recent epidemiological studies, random amplified polymorphic DNA (RAPD) PCR has been extensively used for molecular characterization of bacteria due to its simplicity, rapidity, sensitivity, reproducibility and low cost. It can determine genetic diversity without prior knowledge of the genome under study (10). This study was aimed to investigate the antibiotic resistance profiles, prevalence and production of different type of β-lactamases (ESBL, AmpC and MBL), class 1 integron, and five major virulence genes (*exo*A, *exo*S, *exo*U, *pil*B, and *nan*1) in clinical isolates of MDR *P. aeruginosa* collected from four main referral hospitals in Shiraz and Kerman cities, in southeast of Iran. The clonal relationship of the MDR isolates was also determined.

**Table 1:** Primers used in present study to detect genes and integrolls

**target genes**	**primer sequence (5** **´→** **3** **´** **)**	**size** **(bps)**	**reference**
*exo*S	F: ATC CTC AGG CGT ACA TCC R: ACG ACG GCT ATC TCT CCA C	328	(17)
*exo*U	F: GAT TCC ATC ACA GGC TCG R: CTA GCA ATG GCA GTA ATC G	3308	(17)
*pil*B	F: TCG AAC TGA TGA TCG TGG R: CTT TCG GAG TGA ACA TCG	408	(17)
*nan*1	F: ACG CTC CGT CCA GCC GGA R: GTC TGG ACG ACG GCG GCA	221	(18)
*exo*A	F: GACAACGCCCTCAGCATCACCAGC R: CGCTGGCCCATTCGCTCCAGCGCT	396	(18)
*bla*_VIM1_	F: CCG ATG GTG TTT GGT CGC AT R: GAA TGC GCA GCA CCA GGA	391	(41)
*bla*_IMP1_	F: CTA CCG CAG CAG AGT CTT TG R: AAC CAG TTT TGC CTT ACC AT	587	(41)
*bla*_NDM_	F: GGT TTG GCG ATC TGG TTT TC R: CGG AAT GGC TCA TCA CGA TC	621	(42)
*i* *nt* *I*1	F: GTT CGG TCA AGG TTC TG R: GCC AAC TTT CAG CAC ATG	923	(41)

**Table 2:** Antibiotic resistant profile of *Pseudomonas aeruginosa *collected from hospitalized patients of four hospitals in Iran based on source of isolation

**source**	**number of isolate**	**number (%) resistant isolates**										
		**PTZ**	**CIP**	**MEM**	**IMI**	**ATM**	**CRO**	**CAZ**	**CPM**	**GM**	**TN**	**AK**
**burn exudate**	53	37(69.8)	34(64.15)	36(67.92)	35(66.03)	41(77.35)	41(77.35)	31(58.49)	37(69.81)	35(66.03)	35(66.03)	33(62.26)
**urine**	48	12(25)	16(33.33)	17(35.41)	17(35.41)	14(29.16)	30(62.50)	13(27.08)	13(27.08)	17(35.41)	15(31.25)	13(27.08)
**sputum**	29	7(24.13)	9(31.03)	11(37.93)	10(34.48)	8(27.58)	18(62.06)	8(27)	8(27.58)	8(27.58)	7(24.13)	6(20.68)
**blood**	23	4(17.39)	1(4.34)	4(17.39)	3(13.04)	4(17.39)	12(52.17)	3(13.04)	4(17.39)	4(17.39)	2(8.69)	1(4.34)
**wound**	22	3(13.63)	6(27.27)	5(22.72)	5(22.72)	7(31.81)	14(63.63)	6(27.27)	5(2.72)	5(22.72)	5(22.72)	5(22.72)
**eye**	14	1(7.14)	1(7.14)	6(42.85)	5(35.71)	5(35.71)	8(57.14)	1(7.14)	1(7.14)	1(7.14)	1(7.14)	1(7.14)
**others**	19	2(10.52)	3(15.78)	7(36.84)	7(36.84)	5(26.31)	11(57.89)	2(10.52)	3(15.78)	3(15.78)	2(10.52)	3(15.78)
**total**	208	66(31.73)	70(33.65)	86(41.34)	82(39.42)	84(40.38)	134(64.42)	64(30.76)	71(34.13)	73(35.09)	67(32.21)	62(29.80)

Amikacin (AK), Tobramycin (TN), Gentamicin (GM), cefepime (CPM), ceftazidime (CAZ), ceftriaxone (CRO), aztreonam (ATM), imipenem (IMI), meropenem (MEM), ciprofloxacin (CIP), piperacillin/tazobactam (PTZ)

**Table 3:** Prevalence of multi-drug resistance, β-lactamases production and class 1 integron among *Pseudomonas aeruginosa* from hospitalized patients of four hospitals of Iran

**source/samples**	**number of isolates**	**% MDR**	**ESBL**	**MBL(in MDRPA)**	***AmpC***	**IMI** **mean MIC (µg/ml)** **(E-test)**	*MBL genes	
							**IMP-1**	**VIM**
**burn exudate**	53	36(68)	46(86.8)	32(89.9)	18(50)	64	29(80)	4(11)
**urine**	48	18(37.5)	32(66.7)	9(50)	5(27.7)	64	14(77)	
**sputum**	29	11(38)	20(69)	6(54.5)	1(9.1)	128	10(90)	-
**blood**	23	4(17.4)	12(52)	1(25)	-	32	4(100)	-
**wound**	22	6(27.3)	15(68)	2(33.3)	3(50)	64	6(100)	-
**eye**	14	3(21.5)	6(42.8)	3(50)	1(33.3)	64	3(100)	-
**others**	19	6(31.5)	14(73.7)	1(16.7)	-	32	5(83)	-
**total**	208	84(40.4)	145(70)	54(64.3)	28(33)	-	71(84.5)	4(4.8)

MDR= multidrug-resistant, MBL= metallo-β-lactamase, ESBL= extended spectrum-β-lactamase, IMI= imipenem, intΙ1= class 1 integrons integrase gene. Value in bracket indicate % in MDR population.*.*NDM* gene was not detected in any isolate. Others = fluids/c, Nose/c, etc

**Figure 1:**
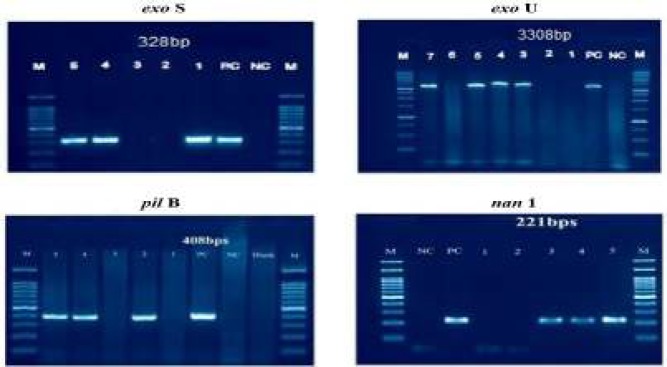
Agarose gel electrophoresis of PCR products of virulent genes

M: marker (100 bp); NC: negative control; PC: positive control

**Figure 2:**
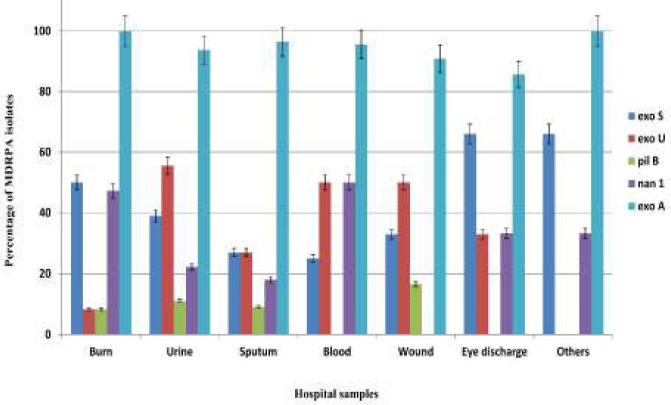
Distribution of *exoS, exoU, pilB, nan1* and *exoA* genes among hospital isolates of MDR *Pseudomonas aeruginosa* from four hospitals in southeast of Iran. The error bar indicates average of three independent experiments

**Figure 3:**
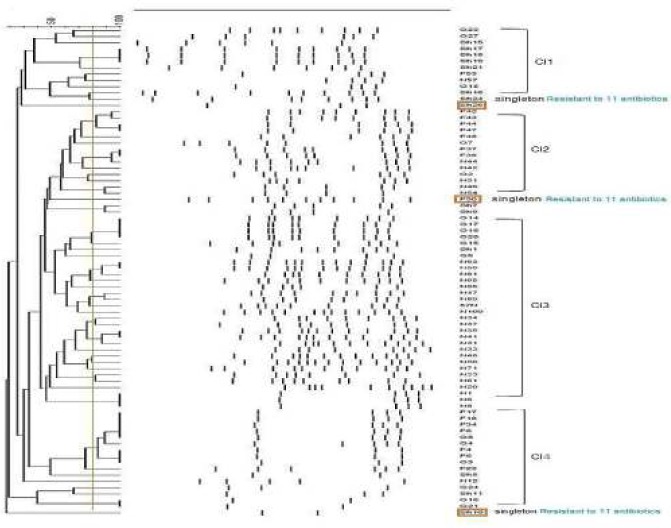
Clonal relationships among *Pseudomonas aeruginosa * isolates recovered from hospitals in two geographic locations in Iran shown by RAPD-PCR fingerprints. Banding patterns were analyzed by the unweighted pair-group method with arithmetic averages (UPGMA) clustering using Gel Compare II software, version 4.0 (Applied Maths, Sint-Matenslatem, Belgium). The vertical lines show 80% similarity cut-off. *N= Namazi, F= Faghihi. G= Ghotbeddin (Shiraz, Iran) and Sh= Shafa hospitals (Kerman, Iran)

## Materials and Methods

***Specimen collection***

From September 2013 to November 2014, a total of 208 non-duplicate isolates of *P. aeruginosa* were recovered from inpatients of four main referral hospitals of A (Namazi with 800 beds), B (Faghihi with 400 beds), C (Ghotbeddin with 80 beds) in Shiraz and D (Shafa with 400 beds) in Kerman, Iran. Samples were taken by expert technicians from hospitalized patients admitted to adult ICUs, NICUs, burn, respiratory care units, general medical and surgical wards, emergency and kidney transplantation wards. The sampling procedure from wound included swabs taken from clinically deep areas of the burn wounds. Blood culture was prepared from patients with suspected bacteremia or sepsis and inoculated into BACTEC™ blood culture medium. The other samples were placed in stuart transport medium (Merck, Darmstadt - Germany) and transferred to the laboratory for identification by standard microbiological tests (11). The bacterial isolates were inoculated into sterile True North TM Cryogenic vials (TNC) containing 1 ml of sterile tryptic soy broth (TSB) (BioMerieux, Marcy-I’Etoile, France), mixed with glycerol (40%) and stored at -70 ^°^C until further examination (11).

***Antimicrobial susceptibility testing***

Kirby–Bauer disk diffusion method was used for antimicrobial susceptibility test as recommended by Clinical and Laboratory Standards Institute guidelines (CLSI-2014) (12). All antibiotic disks were purchased from Mast Co Ltd, UK and used as per manufacturer’s instructions. The following disks were used for antibiotic sensitivity assay (µg per disk); amikacin (AK 30), tobramycin (TN 10), gentamicin (GM 10), cefepime (CPM 30), ceftazidime (CAZ 30), ceftriaxone (CRO 30), aztreonam (ATM 30), imipenem (IMI 10), meropenem (MEM 10), ciprofloxacin (CIP 5), piperacillin/tazobactam (PTZ 110), polymyxin B (PB 300 units) and colistin (CL 10). Minimum inhibitory concentration (MIC) for imipenem resistance isolates was checked by E-strip test (Liofilchem Co., Italy) as CLSI guideline (13).* P. aeruginosa* ATCC 27853 was used as the quality control strain for antimicrobial susceptibility testing throughout this study.

***Identification of ESBL and MBL enzymes by phenotypic methods***

ESBL production was determined by double-disk synergy test (DDST), according to CLSI guidelines and manufacturer’s protocol (Mast^TM^ extended spectrum β-lactamase set; CPD10) (12). Cefotaxime (30 μg) and ceftazidime (30 μg) disks alone or in combination with clavulanic acid (30/10 μg) were used for ESBL detection. An increase in zone diameter of ≥5mm around the disks containing clavulanic acid indicated production of ESBL by the test organism. To counteract the effect of high-level expression of the naturally produced AmpC-type β-lactamase, double**-**disk synergy test was also performed on cloxacillin (200 μg/ml) containing plates (14). Detection of MBL was carried out by MBL-strips (Liofilchem Co, Italy). Briefly, a strip containing imipenem (4-256 µg) on one side and imipenem+EDTA (1-64 µg) on the other side (IMI/IMD) was placed on a Muller Hinton agar plate inoculated with the test organism. The tests were considered positive when the ratio of IMI/IMD was ≥ 8 mm. The results were confirmed by modified Hodges test (15).

***Identification of AmpC by phenotypic method***

Production of AmpC by the isolates was determined using Muller- Hinton agar (BioMerieux, France) plates, inoculated with *Esherichia coli* ATCC 25922 cefoxitin-susceptible strains. A cefoxitin disk (FOX 30 µg) was placed between two blank disks each containing 1:1 mixture of saline and Tris-EDTA 100 × solutions. The paper blank was inoculated with *P. aeruginosa *test isolates. Flattening of the growth inhibition zone toward the paper disk indicates AmpC production (16).

***Detection of MBL resistance genes and class 1 integron by PCR***

The presence of MBL genes, VIM, IMP, NDM and class 1 integrons (*IntΙ1*) was assessed by PCR using specific sets of forward and reverse primers, as shown in Table 1. The bacterial cells were grown overnight on Luria-Bertani (LB) agar. The whole genomic DNA was extracted from single colonies using the DNA genomic extraction kit (Thermo Scientific, Vilnius, Lithuania) and used as a template for PCR amplification, as described by the manufacturer. PCR reaction mixture**s** (25 µl) consisted of 2.5 units of *Taq* DNA polymerase (Thermo Scientific Co), 10 pmol of each primer, 1 µl of dNTP mix (Thermo Scientific Co), 2 µl of DNA template in the PCR buffer provided by manufacturer (Cinnagen, Iran). DNA amplification was conducted in a temperature gradient thermal cycler (Applied Biosystems, 96 well, Veriti, USA). The PCR was as follows; one cycle pre-denaturation at 94 ^°^C for 5 min, 35 cycles of denaturation at 94 ^°^C for 30 sec, annealing at 55 ^°^C for 30 sec (for *int*1and *bla*_IMP-1_ gene temperature was 50 ^°^C), and extension at 72 ^°^C for 30 sec followed by a final extension at 72 ^°^C for 10 min. PCR products (10 µl ) were subjected to gel electrophoresis using 1% agarose gel (Merck, Germany) for 1 hr, stained with UV illuminating dye (Gel Red) and visualized by a UV-gel documentation system (Gel logic200, Kodak, USA) (17).

***Detection of major virulence genes by PCR***

Individual colonies of* P. aeruginosa* isolates were used for detection of *exoA,* *exo*S, *exo*U, *pil*B, and *nan*1 genes using the primers listed in Table 1, as described previously (9, 17, 18).

***Clonal relationships among MDR isolates by RAPD-PCR***

RAPD-PCR was used RAPD primers 208 (5´-ACGGCCGACC-3´) and 272 (5´- AGCGGGCCAA- 3´), as described earlier (19). The amplification protocol consisted of 25 µl RAPD- PCR mixture buffer (10 mM Tris/HCl, 50 mM KCl, 2.5 mM MgCl_2_, pH 8.3) containing 250 µM of each dNTP, 40 pmol oligonucleotide, 1 U* Taq* DNA polymerase Invitrogen (Invitrogen, CA, USA) and 40 ng template DNA. A negative control contained all the components except template DNA. PCR products were separated in 1.5% agarose gels and the resulting band patterns were analyzed using unweighted pair-group method with arithmetic averages (UPGMA) clustering (Gel Compare II software, version 4.0, Applied Maths, Sint-Matenslatem, Belgium). Isolates with 96% or greater similarity were considered as identical, and a cut-off value of 80% similarity was used for clustering.

***Statistical analyses***

All analyses were performed using SPSS, version 16.0 (SPSS Inc, Chicago, IL, USA, 2014). Chi-square test was used to investigate the differences between distributions of categorical data. Two-tailed; *P-values* of ≤ 0.05 were considered statistically significant.

## Results

***Sample sources and antibiotic susceptibility***

One hundred isolates were from hospital A (31% from urine as the most frequent), 55 from hospital B (44% from sputum of patients with respiratory tract infection as the most frequent), 27 were from hospital C (all from burn exudates) and 26 from hospital D (all from burn exudates) and antibacterial susceptibility profiles of the *P. aeruginosa* isolates to 13 antibiotics are presented in Table 2. All isolates were susceptible to polymyxin B and colistin. Overall, 40.4% (n=84) of the isolates were MDR and 40% were carbapenem-resistant. From 84 MDRPA isolates, 53 (63%) were resistant to 11 antibiotics, of which 36 isolates were recovered from burn exudates. The rate of MDR *P. aeruginosa* (defined as resistance to at least 3 antibiotic classes) was 40.4% (in 208 isolates), including 68% from burn exudates, 37.5%, from urinary tract infection and 17.4% from blood samples.

***Prevalence of AmpC, ESBL, MBL and class 1 integrons***

The rates of ESBL, AmpC and MBL production among the MDRPA are shown in Table 3. The highest rate of AmpC production was detected in isolates recovered from burn exudates of hospitals C and D (n=18, 50%). MBL activity was detected in 64.3% (n=54) of the MDR isolates, and *bla*_IMP-1 _and *bla*_VIM_ genes were detected in 84.5% (n=71) and 4.8% (n=4) of these isolates, respectively. No *bla*_NDM_ was detected in this study. In addition, class 1 integrons was detected in 95% (n= 80) of the MDR isolates.

***Detection of virulence genes***

The *exoA* gene was detected in all 208 isolates and was the most frequent gene in both MDR and non MDR *P. aeruginosa *isolates with a total frequency of 98.8% and 93.5%, respectively. The second most frequent virulence gene in the 84 MDR isolates was *exoS* (44%), followed by *nan1* (33.3%), *exoU* (26%) and *pilB* (8.3%) (Figures 1 and 2).

***Clonality relationship among P. aeruginosa MDR isolates***

Based on RAPD-PCR profiles, the number of bands ranged from 10 to 19 with sized from 200–2000 bps. As shown in Figure3, four clusters were identified 80% similarity with 95% identical band patterns, indicating similar genetic backgrounds. We also found two small clusters (two member isolates) and three singletons. Most of the cluster 1 isolates were from hospital D in Kerman and most of cluster 2 isolates were recovered from hospitals A (36%), B (50 %) and C (14 %) in Shiraz.

## Discussion

MDRPA has emerged as one of the most frequently observed nosocomial infectious agents causing a high rate of mortality among hospitalized patients (20). According to European Centre for Disease Prevention and Control 2012, point prevalence survey of healthcare-associated infections, it accounted for 8.9% of nosocomial infections, and 17.4% of lower respiratory tract infections in European acute care hospitals (21).

In the present study, and the rate of MDR *P. aeruginosa* was 40.4% and was highest in the isolates obtained from burn exudates (68%), followed by urinary tract infection isolates (37.5%) and blood samples (17.4%). A report from a surveillance study of 65 laboratories in the United States (1998 to 2001) showed that 7.0% of clinical isolates of *P*. *aeruginosa* from non-ICU patients and 9.1% of isolates from ICU patients were MDR. In another surveillance study conducted on MDRPA in an urban tertiary-care teaching hospital in USA in 2002, it was shown that the rate of MDR was 32% and increased by more than 20% over a five-year period (22). In another investigation in a tertiary care hospital in India from February 2012 to January 2013, the rate of MDRPA was 41.3% which is similar to our results (23). Overall, the rates of resistance of our isolates recovered from burn exudates were significantly higher than that of the other sources (*P*<0.05). Similar rates have been reported from other centers in Iran including 45.3% among burn patients studied in 2012 in Guilan by Nikokar *et al*. (24), and 45% by Fazeli *et al*. (25) in 2017 from patients at the university teaching hospital in Iran. Higher rates of MDRPA were reported by Yousefi *et al*. (2011-2012) in Shiraz (62.8%) and Ghanbarzadeh *et al*. (2015) from a single center for burn patients in Tehran, Iran (93.1%) (26, 27)**.**

In our study, burn isolates exhibited the highest degree of resistance (>60%) to 11 antibiotics compared to the isolates from other sources (Table 2). Imipenem resistance was detected in 84.5% of (71 out of 84) MDRPA isolates and 39% (82 out of 208) of all *P. aeruginosa* isolates. As observed, compared to the isolates from other sources MDRPA isolated from burn exudates had the highest rate of ESBL (86.8%), MBL (89.8%) and AmpC (50%) production (Table 3).

In a report from burn patients in Hospital C in 2006, the rate of ESBL production was 4.3%, AmpC was 11.4% and MBL was 0% (28). Excessive use of antibiotics could be associated with the development of more resistant strains as a result of and horizontal gene spread. High frequencies of ESBL production in *P. aeruginosa* isolates from burn patients were reported from Iran by Rafiee *et al*. (39.2% ESBL, 37.3% MBL, and 68.6% AmpC production among 51 MDRPA isolates) (29)_._ In a research carried out by Salimi and Eftekhar on 128 imipenem-resistant isolates of *P. aeruginosa* in Tehran, Iran, 12.5% were capable of producing ESBL, 25% MBL, and 81% produced AmpC β-lactamases (30). MBL* bla*_VIM_ and* bla*_IMP-1_ genes are the common in MDR isolates of *P. aeruginosa* in Iran (31-33). The rate of *bla*_IMP-1_ gene in our study was higher than those reported from other parts of Iran. Also, 95.2% of our MDRPA isolates had *IntI1* gene which is similar with the study of Khosravi *et al.* from Ahvaz and Yousefi *et al.* from Tehran (6, 34). However, lower rate of *IntI1* gene were reported in other parts of Iran (1, 24, 34). Investigating the three MBL genes, *bla*_VIM_, *bla*_IMP-1_ and *bla*_NDM_ among MDRPA strains, revealed that all imipenem-resistant isolates harbored *bla*_IMP-1_ gene. The common MBL genes in Asia are *bla*_VIM_ and* bla*_IMP-1_ (31). A higher rate of *bla*_IMP-1_ gene was detected in the present study in comparison with other Iranian studies: Shahcheraghi *et al*. 0% in 2010, Kalanter *et al*. 3% in 2011 and 2012, and Sarhangi *et al*. 9.75% in 2012 (31, 35, 36). The rate of* bla*_VIM_ gene was 4.8% detected in 4 MDRPA strains isolated from burn exudates, higher than similar studies from Iran (31, 37). In addition, recently our group identified one strain of *P. aeruginosa *harboring the *bla*_NDM_ gene in Kerman, Iran (unpublished result).

Presence of diverse virulence genes in *P. aeruginosa* hospital isolates has been shown to associate with the intensity and severity of infections (38). The high worldwide prevalence of exotoxin A in *P.* *aeruginosa* has resulted in its use for identification of clinical isolates (39). The prevalence rates of *exo*S and *nan*1 genes were significantly higher in burn exudates (50% for* exo*S and 47% for* nan*1, respectively) than in other samples (*P*=0.0001). In contrast, the prevalence of *exo*U gene was significantly lower in burn exudate (16%) (*P*=0.023). The least virulence gene detected in this study was* pil*B (n=7, 8.3%), similar in burn exudates and non-burn samples. The higher frequency of *nan*1in burn patients may be due to its neuraminidase activity which facilitates bacterial attachment to the epithelial surfaces of burns and airways of cystic fibrosis patients resulting in colonization of *P. aeruginosa *(38).

There are few reports about clonal relationship among hospital isolates of *P. aeruginosa* in Iran. Our analysis of RAPD data showed four large clusters consisting of 77 isolates out of 84 (91.7%). We also detected the aggregation of isolates related to specific clusters in different hospitals (Figure 3). The high genetic similarity among MDRPA isolates suggests the cross-acquisition of infection. The dendrogram analysis revealed that cluster 3 exhibited the largest fingerprint similarity consisting of 29 isolates. Among the cluster 3 isolates, 76% were from hospital A and 21% from hospital C (Figure 3). Members of this cluster demonstrated close genetic relationships compared to other clusters, suggesting the possible spread of cluster 3 clones in different wards of hospitals A and C in Shiraz. Most members of cluster 4 were from hospitals B and C. As presented in Figure 3, all singletons showed resistance to 11 antibiotics, simultaneously.

Taheri *et al*. studied the genetic similarity among 73 *P. aeruginosa* isolates from Tehran referral hospitals by RAPD patterns and showed 67 different patterns, each containing 2-3 isolates, mostly from ICU (40). They concluded that most of the isolates were probably originated from the host.

## Conclusion

Based on the results, it can be concluded that our *P. aeruginosa* hospital isolates are highly resistant to different classes of antibiotics and sensitive to colistin and polymyxin B, which could be used as an empirical therapy in critically ill patients, especially in burn patients and those admitted to ICU. High genetic similarity among MDRPA isolates indicates cross-acquisition of infection, suggesting the importance of infection control in decreasing the prevalence of MDRPA in hospitals.
